# A Rapid *In Situ* Colorimetric Assay for Cobalt Detection by the Naked Eye

**DOI:** 10.3390/s16050626

**Published:** 2016-05-02

**Authors:** Sung-Min Kang, Sung-Chan Jang, Gi Yong Kim, Chang-Soo Lee, Yun Suk Huh, Changhyun Roh

**Affiliations:** 1 Biotechnology Research Division, Advanced Radiation Technology Institute (ARTI), Korea Atomic Energy Research Institute (KAERI), 29 Geumgu-gil, Jeongeup, Jeonbuk 56212, Korea; smk@kaeri.re.kr (S.-M.K.); jsc@kaeri.re.kr (S.-C.J.); kgy@kaeri.re.kr (G.Y.K.); 2 Department of Chemical Engineering, Chungnam National University, 99 Daehak-ro, Yuseong-gu, Daejeon 34134, Korea; 3 Department of Biological Engineering, Biohybrid Systems Research Center (BSRC), Inha University, 100 Inha-ro, Incheon 22212, Korea; 4 Radiation Biotechnology and Applied Radioisotope Science, University of Science and Technology (UST), 217 Gajeong-ro, Daejeon 34113, Korea

**Keywords:** cobalt, colorimetric, chemosensor, on-site detection, naked eye

## Abstract

A simple, rapid, and convenient colorimetric chemosensor of a specific target toward the end user is still required for on-site detection and real-time monitoring applications. In this study, we developed a rapid *in situ* colorimetric assay for cobalt detection using the naked eye. Interestingly, a yellow to light orange visual color transition was observed within 3 s when a Chrysoidine G (CG) chemosensor was exposed to cobalt. Surprisingly, the CG chemosensor had great selectivity toward cobalt without any interference of other metal ions. Under optimized conditions, a lower detection limit of 0.1 ppm via a spectrophotometer and a visual detection limit of 2 ppm with a linear range from 0.4 to 1 ppm (R^2^ = 0.97) were determined. Moreover, the CG chemosensor is reversible and maintains its functionality after treatment with chelating agents. In conclusion, we show the superior capabilities of the CG chemosensor, which has the potential to provide extremely facile handling, high sensitivity, and a fast response time for applications of on-site detection to real-time cobalt monitoring for the general public.

## 1. Introduction

The highly sensitive and selective determination of metal ions (e.g., light, heavy, rare, precious, and alloys, *etc.*) has attracted significant interest owing to their important role in the biological and environmental fields [1]. Recently, many techniques have been reported for the detection of heavy metal ions owing to their significant impacts on human beings and the environment [2]. In particular, as an important but harmful heavy metal ion, cobalt is a relatively rare element that is used in various products such as supercapacitors [3], magnets [4], alloys [5], pigments [6], metal finishing [7], mining [8], catalysts [9] and lithium-ion battery manufacturing [10], because of its specific hardness and resistance to oxidation [11,12]. Although cobalt is used as a popular industrial material, its unregulated exposure causes serious detrimental effects including alarms and asthma, cardiac and thyroid damage, heart failure and heart disease, and elevated red blood cells [13,14,15,16]. In addition, other major sources of cobalt in the environment are soil, dust, seawater, and forest fires [17]. Moreover, it is released from burning coal and oil, vehicle and airplane exhaust, diamond polishing, and chemical and hard metal industries [18]. Hence, the development of efficient methods for on-site and real-time monitoring is crucial to detect cobalt in the environment for protecting human health.

Conventional methods, such as surface-enhanced Raman scattering spectroscopy [19], inductively coupled plasma mass spectrometry [20], inductively coupled plasma atomic emission spectrometry [21], fiber optic-linear array detection spectrophotometry [22], flame atomic absorption spectroscopy [23,24,25], and electrochemical sensors [26,27], have been reported for the detection of cobalt. However, these methods require expensive sophisticated instruments, tedious sample preparation procedures, time, and well-trained experts. Moreover, the major disadvantage is that conventional methods are unsuitable for on-site detection with real-time monitoring.

Colorimetric methods have their own advantages such as simplicity, high sensitivity and selectivity, and a reasonable response time [28,29,30,31]. In particular, these methods, which can be conveniently and easily monitored by the naked eye, are appropriate for real-time monitoring of target heavy metal ions and potential application in on-site detection owing to their simplicity and portability [32]. To date, several approaches are reported, such as chemiluminescence [33], electro chemiluminescence [34], and fluorescein probes [35,36,37]. In particular, a number of colorimetric sensors based on functional gold and silver nanoparticles (NPs) have been reported [38,39,40]. The nanoparticles show excellent selectivity and sensitivity as a colorimetric sensing probe. In particular, gold nanoparticles offer excellent localized surface plasmon resonance (LSPR) properties, exhibiting a well-defined color, and easy visualization based on color changes between the dispersed and aggregated nanoparticles [41]. However, there are still many things (e.g., nanoparticle size and shape control, experimental conditions for ligand activation, and stabilizers) to consider when detecting target materials [42,43].

In this paper, we present a rapid *in situ* colorimetric assay for cobalt in an aqueous solution. Interestingly, the interaction between a Chrysoidine G (CG) chemosensor and cobalt induces a color transition from yellow to light orange. Therefore, the feasibility for a sensitivity, selectivity, and rapid assay of cobalt using a CG chemosensor has been extensively demonstrated. Furthermore, we developed a reversible color “on-off” system using an external chelating agent for real-time on-site detection. The proposed colorimetric assay shows great potential for the simple, easy, and quickly responsive on-site detection of cobalt.

## 2. Materials and Methods

### 2.1. Chemicals

4-Phenylazo-*m*-phenylenediamine (Chrysoidine G, CG), lithium chloride, iron(II) chloride tetrahydrate, iron(III) chloride hexahydrate, magnesium(II) chloride hexahydrate, manganese(II) chloride tetrahydrate, and aluminum(III) chloride hexahydrate was purchased from Sigma-Aldrich Chemicals (St. Louis, MO, USA). Cobalt standard solutions were purchased from Kanto Chemical Co., Inc. (Tokyo, Japan). Standard copper, zinc, arsenic, cadmium, and mercury solutions were purchased from CPI International, Co. (Santa Rosa, CA, USA). All reagents and chemicals were of analytical grade and were prepared using highly pure water with a resistivity of 18 MΩ·cm.

### 2.2. Preparation of CG Aqueous Chemosensor and Detection of Cobalt

The CG (180 mg) was dissolved in water (100 mL) and was diluted to double-distilled water to make a final concentration of 7 × 10*^−^*^5^ M. The standard cobalt solutions were then adjusted to a CG aqueous chemosensor and shaking gently for a three seconds. After the reaction, we checked the color change by the naked eye and recorded the UV-vis spectra on an Infinite^®^ UV·M200 spectrometer (TECAN, Salzburg, Austria), using a 96-well plate for the measurements.

### 2.3. Optimization of Suitable Conditions for Colorimetric Detection

To examine the effect of pH, the desired pH solution was prepared by adjusting 1 N NaOH or 1 N HCl. The pH of the solution was measured using a SevenCompact™ pH/ion S220 meter (Mettler Toledo Instruments Co., Greifensee, Switzerland). Moreover, various concentric CG chemosensors were prepared to determine the initial concentration of the CG chemosensor (7 × 10*^−^*^4^ M, 7 × 10*^−^*^5^ M, and 7 × 10*^−^*^6^ M).

## 3. Results and Discussion

### 3.1. Selective Recognition Study for CG Chemosensor

**Figure 1:**
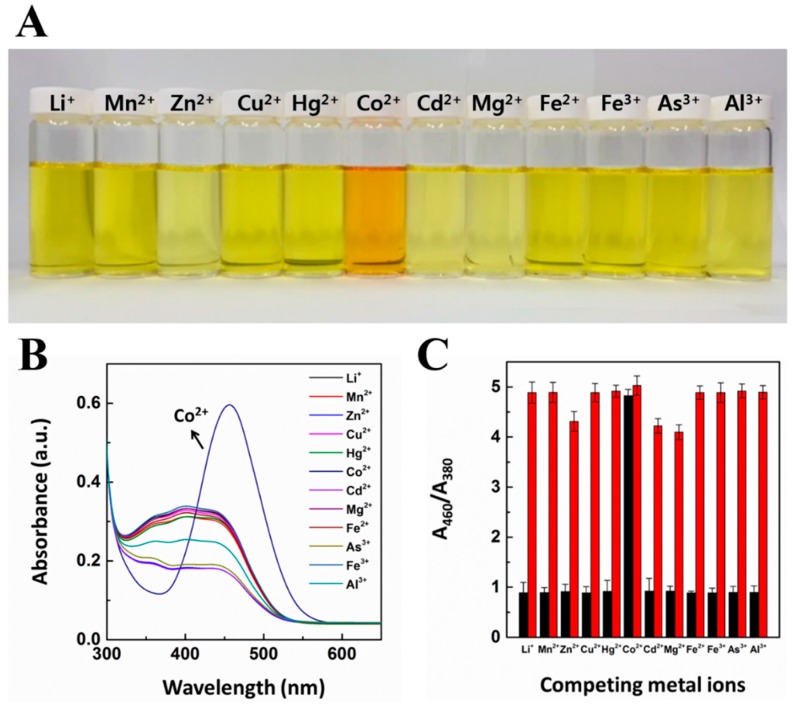
(**A**) Photographs for color changes of CG chemosensor upon addition of various metal ions under visible light; (**B**) UV-vis absorbance spectra of CG chemosensor upon the addition of various metal ions in solution; (**C**) High selectivity toward the cobalt ions and Absorbance responses of CG containing Co^2+^ with the other competing metal ions. The concentration of CG chemosensor and cobalt are 7 × 10^−5^ M and 2 ppm, respectively. Each experiment was conducted three times.

To examine the detection behavior of the CG chemosensor in water, the visible color and UV-vis absorbance spectra upon exposure to various metal ions were recorded. The ability of selective recognition toward cobalt was demonstrated by considering physiologically and environmentally relevant metal ions as their nitrate salts. As shown in Figure 1A, the CG chemosensor shows almost no change in color in the presence of Li^+^, Mn^2+^, Zn^2+^, Cu^2+^, Hg^2+^, Cd^2+^, Mg^2+^, Fe^2+^, Fe^3+^, As^3+^, and Al^3+^, whereas the presence of Co^2+^ exhibited a color change from yellow to light orange (each of them was added at 2 ppm). Figure 1B presents a selective cobalt detection UV-vis absorbance spectrum. This result clearly shows that various metal ions without cobalt did not show any significant response to the CG chemosensor. In addition, the results represented that the UV-vis absorbance ratio toward cobalt was significantly higher than for the coexistent metal ions (Figure 1C). The color transition phenomena occurred in the presence of 2 ppm of cobalt in each cation-CG chemosensor mixed aqueous solution. The quantitative nature for the selective detection of cobalt by CG chemosensor is described in Figure 1C. The distinct relative absorbance ratio of cobalt might be the cause for the distinct light orange color of the CG chemosensor containing cobalt. Interestingly, this result implies that a CG chemosensor can serve as a potential candidate for “naked eye” cobalt detection in aqueous systems.

### 3.2. The Effect of pH and CG Chemosensor Concentration

**Figure 2:**
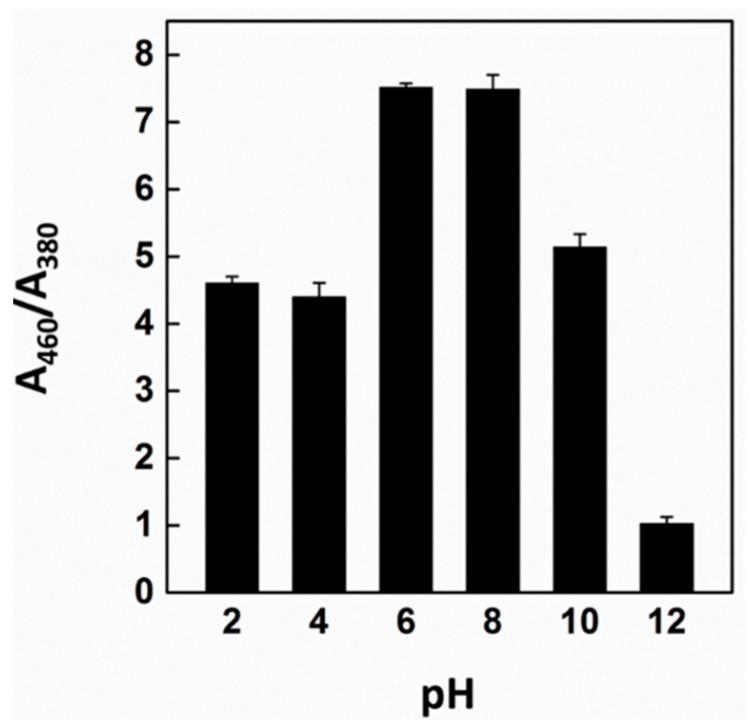
The color changes of CG-Co^2+^ complex at different pH. UV-vis analysis at pH conditions for colorimetric detection of cobalt. The concentration of cobalt is 2 ppm. Each experiment was performed three times.

Further experiments were conducted by various essential factors such as the pH of the aqueous solution and the initial concentration of the CG chemosensor. First, to determine the optimized pH for an efficient colorimetric detection performance, experiments were performed in a pH range of 2–12, the results of which are shown in Figure 2. The color change performance of the CG-Co^2+^ complexation occurred within the range between pH 6 and 8, while its color was maintained in the original state at pH 2, 4, 10, and 12. This result indicates that cobalt can be clearly detected by the naked eye, and UV-vis absorbance measurements using the optimized condition within a pH range of 6–8 (Figure 2).

In addition, the optimum concentration of the CG chemosensor was investigated to improve the visibility in an aqueous detecting system. In this regard, the initial condition of the CG chemosensor at different concentrations was demonstrated through simple naked eye monitoring and a UV-vis absorbance analysis. As shown in Supplementary Figure S1A, in a relatively high (1) and low (3) concentrated aqueous solution of CG chemosensor, the addition of cobalt can cause a slight enhancement of the absorbance ratio, but only 7 × 10^−5^ M (2) can induce a remarkable color change from yellow to light orange. In addition, the UV-vis absorbance analysis clearly showed the difference in color intensities between before and after CG-Co^2+^ complexation (Supplementary Figure S1B). Although a higher concentration of the CG chemosensor was used to increase the sensitivity for cobalt, it lacks a difference in color transition for recognition by the naked eye. In addition, in the case of a lower concentration of the CG chemosensor, the color transition did not appear. This result indicates that the balance between the sensing probe and specific target is an essential parameter in a colorimetric naked eye system. Thus, a CG chemosensor concentration of 7 × 10^−5^ M was used.

### 3.3. Stoichiometric Binding Study of CG-Co^*2*+^ Complex

**Figure 3:**
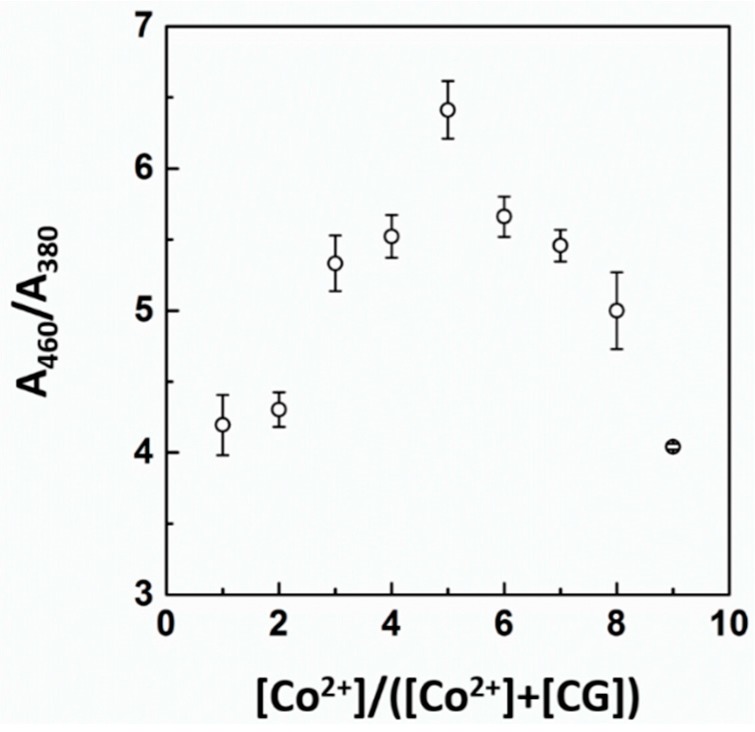
Job’s plot obtained between CG chemosensor and cobalt. The total concentration of the CG chemosensor and cobalt was 10 μM. Each experiment was conducted three times.

To determine the stoichiometry between a CG chemosensor and cobalt ions, a Job’s plot experiment was carried out (Figure 3) [44]. The stoichiometry of binding between the CG chemosensor and cobalt was determined by keeping the sum of the initial concentrations of the CG chemosensor and cobalt constant at 10 μM and varying the molar ratio of Co^2+^ (*X_m_* = ([Co^2+^]/([Co^2+^] + [CG])). By following the change in absorbance ratio (A_460_/A_380_), the maximum absorbance ratio of the CG-Co^2+^ complex was achieved at a mole fraction of approximately 50% of the cobalt ions. This result suggests that the stoichiometry of binding of the CG chemosensor with cobalt ions is 1:1. Based on the stoichiometry study, we estimate that the complexation between the CG chemosensor and cobalt can be attributed to the hydrated cobalt size and the entropic free volume and spatial arrangement of azobenzene and the amino groups of Chrysoidine G [45,46]. Thus, a proposed mechanism of CG-Co^2+^ binding can be presented (Supplementary Figure S2).

### 3.4. Response Time Monitoring for CG-Co^*2*+^ Complexation

**Figure 4:**
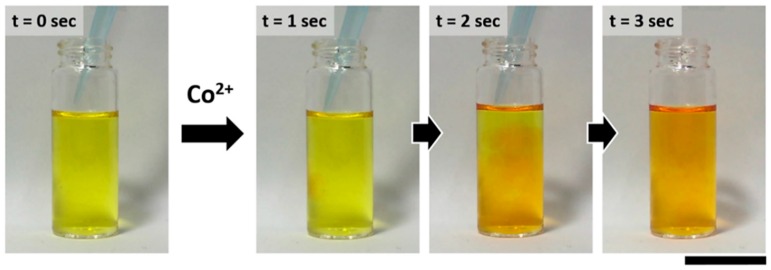
Time sequence images of aqueous CG chemosensor in the presence of cobalt. The concentration of CG chemosensor and cobalt are 7 × 10^−5^ M and 2 ppm, respectively. The scale bar is 3 cm.

A fast response time is an important factor in analytical sensing applications for real-time monitoring. To envision a real application, we conducted a real-time imaging experiment of the reaction between the CG chemosensor and cobalt. Interestingly, the color transition of an aqueous CG chemosensor solution occurred within a few seconds in the presence of cobalt ions, as shown in Figure 4. When 5 ppm of cobalt ions were added to an aqueous CG chemosensor, it gradually changed from yellow to light orange within 3 s. These visible results suggest that the CG chemosensor can be applied to the real-time monitoring of a portable indicator with simple and rapid ‘naked eye’ detection of cobalt.

### 3.5. UV-Vis Titration Study for CG Chemosensor

**Figure 5:**
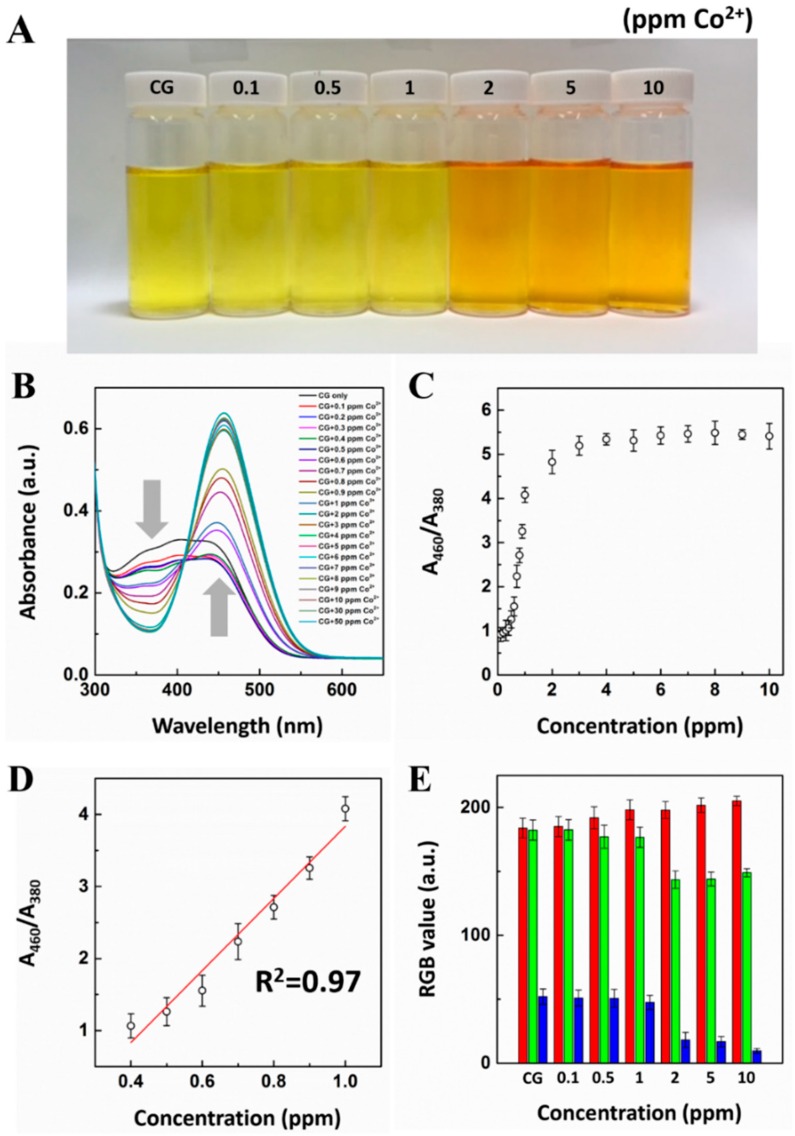
Colorimetric titration of cobalt. (**A**) Photographs for the color change of CG chemosensor to the concentrations of cobalt; (**B**) UV-vis absorbance changes of CG chemosensor in the presence of a serial concentration of the cobalt; (**C**) Intensity ratio (A_460_/A_380_) *versus* the different concentrations of cobalt ion added; (**D**) Linear plot of cobalt concentration based on UV-vis absorbance analysis; (**E**) Quantitative analysis for RGB color profile of CG-Co^2+^ complexationss. The concentration of CG chemosensor and cobalt are 7 × 10^−5^ M and 2 ppm, respectively.

To evaluate the sensing performance toward cobalt, we performed a colorimetric titration experiment with different concentrations of cobalt ranging from 0.1 ppm to 50 ppm. As shown in Figure 5A, a color transition can be observed when the concentration of cobalt is beyond 2 ppm by the naked eye. In detail, we conducted a UV-vis titration experiment to demonstrate the absorbance change for a precise response of a CG chemosensor toward cobalt ions (Figure 5B). Interestingly, the absorbance peak is clearly red-shifted at 460 nm, and a peak at 380 nm gradually decreased with an increases in cobalt concentration. Meanwhile, one clear isosbestic point appeared at 410 nm, indicating that the well-defined point is a clear interconversion between the complexed and uncomplexed forms that occur. It can also be explained that the CG chemosensor formed CG-Co^2+^ chelate bonds between the CG chemosensor and cobalt ions. The absorbance intensity ratio (A_460_/A_380_) as a function of cobalt concentration is shown in Figure 5C. The saturation of the absorbance intensity ratio was reached with an increase in cobalt concentration at 5 ppm of cobalt ions. The CG chemosensor exhibited a linear range of detection for cobalt from 0.4 ppm to 1.0 ppm. In addition, the plot of A_460_/A_380_ against various cobalt concentrations presented a good linear relationship (R^2^ = 0.97), where A_460_ and A_380_ are the UV-vis absorbance intensities in the presence of cobalt (Figure 5D). 

Also, numerical data processing was performed using digital images taken with a smartphone. As shown in Figure 5E, numerical RGB values of the colorimetric images were extracted by using ImageJ software [47]. Interestingly, the CG chemosensor exhibited high response toward increasing cobalt concentrations in terms of decreased values in Green (G) and Blue (B) compared to increased values in Red (R). Of note, cobalt could be detected by the naked eye by the colorimetric response of the CG chemosensor with a detection limit as a 2 ppm. In addition, an advantage of our colorimetric system is that it can be operated in pure water. A real sample was collected from the Korea Atomic Energy Research Institute (KAERI). Interestingly, as shown in Supplementary Figure S3, the CG chemosensor showed no color transition for a real sample (sample A). However, on-site detection could be performed by simply introducing an artificial waste sample (sample B, including 2 ppm Co^2+^) and observing the resulting color change from yellow to light orange. This result suggests that the CG chemosensor test results can be easily confirmed by the naked eye, even when the cobalt ions are contaminate by unknown samples.

### 3.6. Reversibility Test

**Figure 6:**
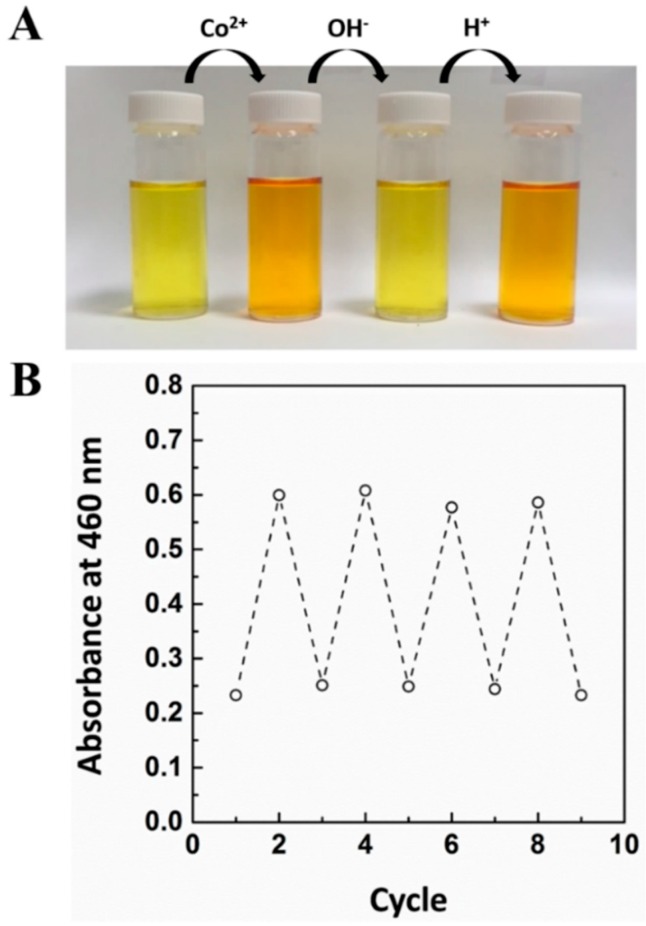
(**A**) Photographs of the reversibility behavior between CG chemosensor and cobalt; (**B**) Continuously repeated UV-Vis absorbance profile during stepwise “on-off” switch reaction. The concentration of CG chemosensor is 7 × 10^−5^ M.

For the on-site reuse of a specific target, the limit of reversibility is important. To understand further the affinity interactions between the CG chemosensor and cobalt in water at the molecular level, externally strong chelating agents such as NaOH were added after the detection of a CG chemosensor response in the presence of cobalt. Reversibility is needed to reuse the CG chemosensor for the detection of the same target. In this regard, a visible color change was observed in the presence of 2 ppm of cobalt followed by the introduction of a 1 N NaOH aqueous solution. Eventually, in the presence of NaOH, the color is changed from light orange to yellow. This result indicates that Co^2+^ can preferentially react with NaOH for deprotonation into a more stable NaOH-Co^2+^ complex in basic medium. Sequentially, the recovery of a light orange color is induced by introducing a 1 N HCl aqueous solution (Figure 6A). 

These results suggest that the acidity or base level of the solution has no effect on the stability of the CG chemosensor. Moreover, this reversible color change procedure was continually repeated, as shown in Figure 6B. As shown in Figure 6, this result led to the development of a molecular level sensory technology signal using an “on-off” absorbance intensity profile.

## 4. Conclusions

In conclusion, we successfully elucidated a rapid colorimetric assay using a CG chemosensor. Importantly, the CG chemosensor exhibited good selectivity and sensitivity toward cobalt, which could be simply confirmed through a color transition phenomenon from yellow to light orange. In addition, we found that the optimal conditions such as the external (≈pH) and internal (≈ initial concentration of CG) factors could be determined by the naked eye and through a UV-vis absorbance measurement. Furthermore, the reversibility of the CG chemosensor was demonstrated through a simultaneous injection of chelating agents. We note that the proposed CG chemosensor with a colorimetric assay exhibits an enhanced on-site and real-time monitoring performance compared to existing methods: (1) visual sensitivity with the naked eye has a limit of detection on the order of 2 ppm; (2) a reasonably rapid response time (<3 s); and (3) excellent selectivity without any interference from other metallic ions. We believe that it is a very simple, convenient and rapid detecting method for cobalt and can be a potential candidate for practical applications such as on-site test kits and real-time monitoring.

## Supplementary Materials

The following are available online at http://www.mdpi.com/1424-8220/16/5/626/s1.
