# Supplementary material for: A Rapid In Situ Colorimetric Assay for Cobalt Detection by the Naked Eye
Source: Sensors (Basel). 2016 May 2;16(5):626. doi: 10.3390/s16050626 (PMC4883317; doi:10.3390/s16050626)
Supplement: Supplementary file 1 [file sensors-16-00626-s001.docx]

Supplementary Materials: A Rapid *In-Situ* Colorimetric Assay for Cobalt Detection by
Naked Eye

Sung-Min Kang, Sung-Chang Jang, Gi Yong Kim, Chang-Soo Lee, Yun Suk Huh and
Changhyun Roh


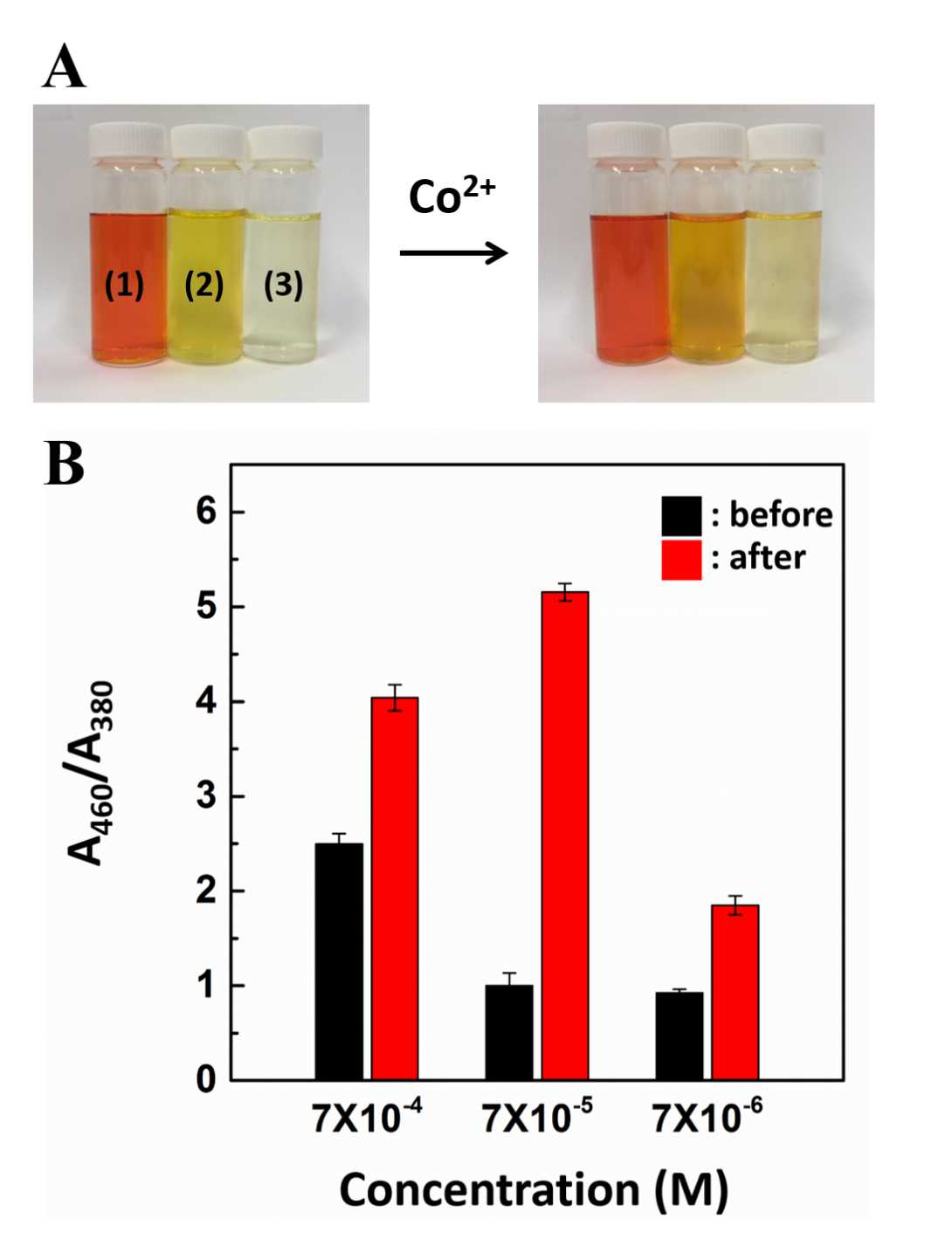


**Figure S1.** (**A**) The effect of initial CG chemosensor concentration for detection of cobalt. The concentrations of CG chemosensor were (**1**) 7 × 10^−4^ M; (**2**) 7 × 10^−5^ M; and (**3**) 7 × 10^−6^ M, respectively; (**B**) The difference of UV-Vis absorbance ratio to compare before with after reaction. The concentration of cobalt is 2 ppm. Each experiment was performed three times.


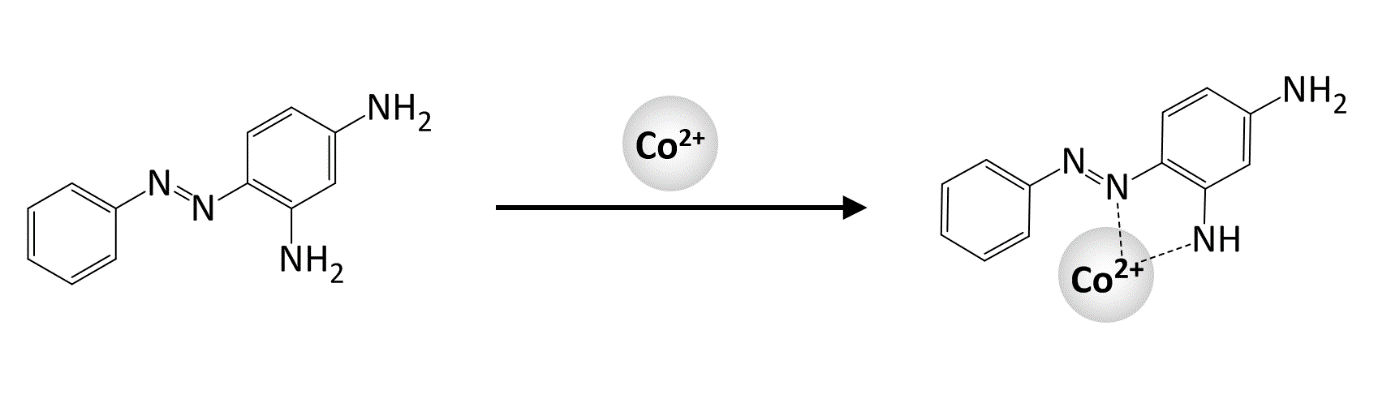


**Figure S2.** Proposed mechanism of CG chemosensor for sensing of Co^2+^.

**
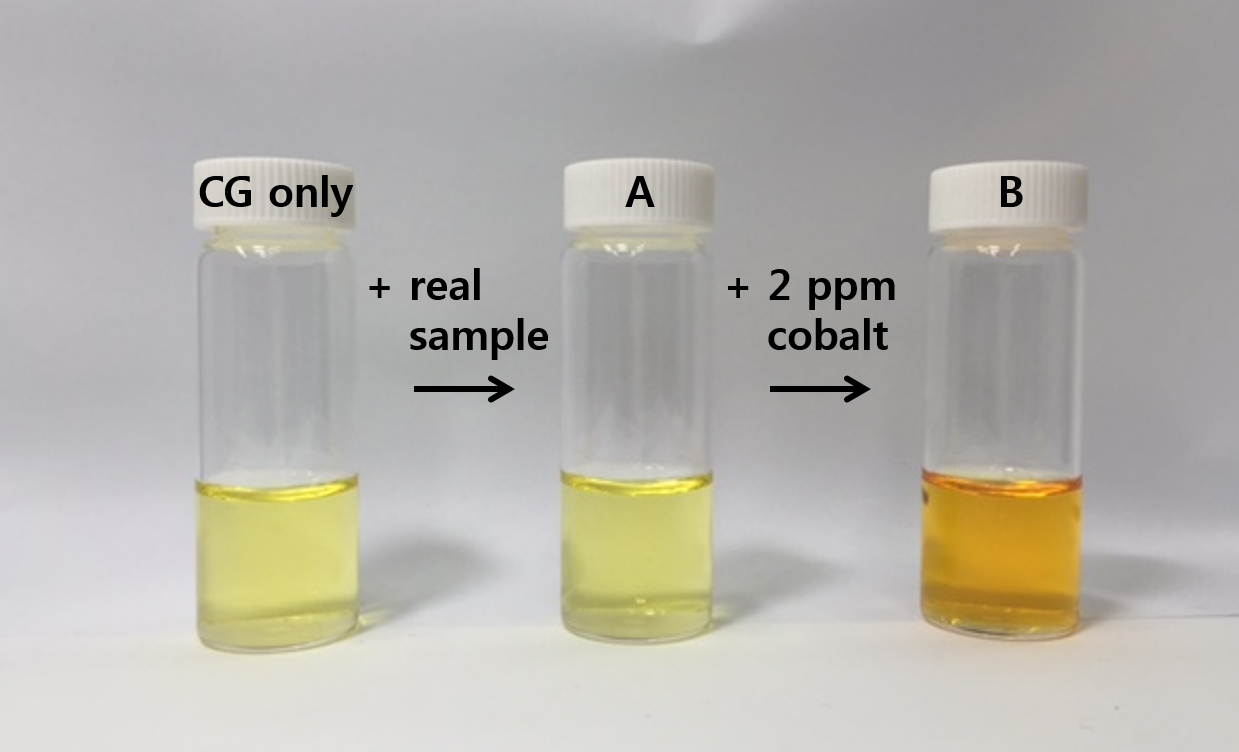
**

**Figure S3.** The simple and on-site colorimetric assay in real samples.
